# Deep-learning: investigating deep neural networks hyper-parameters and comparison of performance to shallow methods for modeling bioactivity data

**DOI:** 10.1186/s13321-017-0226-y

**Published:** 2017-06-28

**Authors:** Alexios Koutsoukas, Keith J. Monaghan, Xiaoli Li, Jun Huan

**Affiliations:** Department of Electrical Engineering and Computer Sciences, University of Kansas, Lawrence, KS 66047-7621 USA

**Keywords:** Deep learning, SARs, Cheminformatics, Machine-learning, Data-mining, Random forest, kNN, Support vector machines, Naïve Bayes

## Abstract

**Background:**

In recent years, research in artificial neural networks has resurged, now under the deep-learning umbrella, and grown extremely popular. Recently reported success of DL techniques in crowd-sourced QSAR and predictive toxicology competitions has showcased these methods as powerful tools in drug-discovery and toxicology research. The aim of this work was dual, first large number of hyper-parameter configurations were explored to investigate how they affect the performance of DNNs and could act as starting points when tuning DNNs and second their performance was compared to popular methods widely employed in the field of cheminformatics namely Naïve Bayes, k-nearest neighbor, random forest and support vector machines. Moreover, robustness of machine learning methods to different levels of artificially introduced noise was assessed. The open-source Caffe deep-learning framework and modern NVidia GPU units were utilized to carry out this study, allowing large number of DNN configurations to be explored.

**Results:**

We show that feed-forward deep neural networks are capable of achieving strong classification performance and outperform shallow methods across diverse activity classes when optimized. Hyper-parameters that were found to play critical role are the activation function, dropout regularization, number hidden layers and number of neurons. When compared to the rest methods, tuned DNNs were found to statistically outperform, with *p* value <0.01 based on Wilcoxon statistical test. DNN achieved on average MCC units of 0.149 higher than NB, 0.092 than kNN, 0.052 than SVM with linear kernel, 0.021 than RF and finally 0.009 higher than SVM with radial basis function kernel. When exploring robustness to noise, non-linear methods were found to perform well when dealing with low levels of noise, lower than or equal to 20%, however when dealing with higher levels of noise, higher than 30%, the Naïve Bayes method was found to perform well and even outperform at the highest level of noise 50% more sophisticated methods across several datasets.

**Electronic supplementary material:**

The online version of this article (doi:10.1186/s13321-017-0226-y) contains supplementary material, which is available to authorized users.

## Background

Machine learning techniques have become an integral part of the modern drug discovery process. Data mining methods based on machine learning techniques are routinely applied to model complex physicochemical and chemo-biological endpoints. Applications range from quantitative structure–property relationships (QSPRs) [1–3], quantitative structure–activity relationships (QSARs) [4, 5] to in silico mode-of-action analysis and predictive toxicology [6, 7].

Artificial neural networks (ANNs) were once popular in the field of molecular informatics and have been applied for wide range of QSARs/QSPRs applications [8–17]. With the introduction of shallow methods such as random forest and support vector machines and technical difficulties associated with ANNs led to gradual decrease of popularity of these methods. In recent years, research in ANNs has resurged, now under the deep-learning umbrella, and grown extremely popular due to major breakthroughs in computing capabilities, attracting considerable attention from both academia and industry. Although reported applications of deep-learning techniques still remain limited in the field of molecular modeling they are fast gaining attention [18]. Two major crowd-sourced competitions, the Merck QSAR competition, held in 2012 at the Kaggle data mining platform, and the Tox21 data-challenge 2014 for chemical risk assessment were both won by groups that utilized deep learning methods as main machine learning techniques [19, 20]. The recent success demonstrates that deep learning methods are well suited for modeling complex biological data to support drug discovery and toxicological research.

ANNs were designed to mimic the efficiency and robustness of biological systems inspired by the complex cerebral cortex to process the myriads amount of sensory data [21]. ANNs attempt to represent highly non-linear and varying functions by combining multiple levels of representations through interconnected non-linear transformations [22]. Deep-learning methods are part of distributed representation-learning algorithms that attempt to extract and organize discriminative information from the data by discovering features that compose multi-level distributed representations [23]. Representation learning algorithms have been applied with success to model complex real-world problems, such as speech recognition, signal processing and image classification [21, 24–26].

**Fig. 1:**
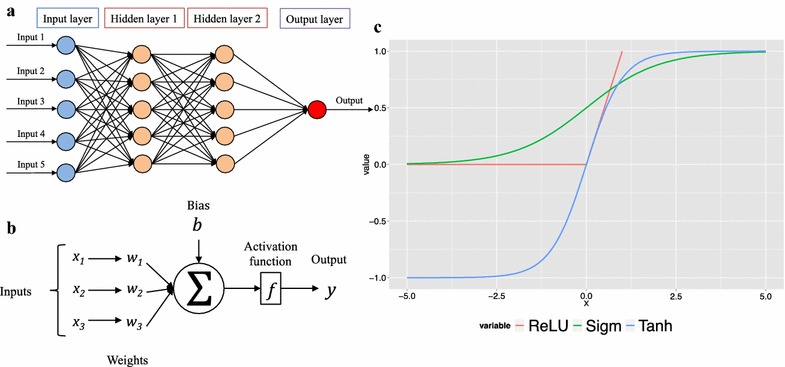
**a** A feed-forward deep neural network with two hidden layers, each layer consists of multiple neurons, which are fully connected with neurons of the previous and following layers. **b** Each artificial neuron receives one or more input signals *x*_1_, *x*_2_,…, *x*_*m*_ and outputs a value *y* to neurons of the next layer. The output *y* is a nonlinear weighted sum of input signals. Nonlinearity is achieved by passing the linear sum through non-linear functions known as activation functions. **c** Popular neurons activation functions: the rectified linear unit (ReLU) (*red*), Sigmoid (Sigm) (*green*) and Tanh (*blue*)

An DNN consists of multiple fully-connected layers: an input layer, one or multiple hidden layers and a single output layer [27]. In feed-forward DNN, one of the mainstream deep learning methods, information is flowing forward from the input layer, through the hidden layers and towards the output layer, illustration shown in Fig. 1a. The building block of DNN is the artificial neuron, which was introduced in 1943 by McCulloh and Pitts [28] and was inspired by biological neurons, illustration shown in Fig. 1b. Each neuron receives one or more input signals *x*_1_, *x*_2_, …, *x*_*m*_ and outputs a value *y* to neurons of the next layer and so forth. The output *y* is a nonlinear weighted sum of input signals, Fig. 1c shows popular activation functions the rectified linear units (ReLU), Tanh and Sigmoid (Sigm).

Deep neural networks are typically trained, by updating and adjusting neurons weights and biases, utilizing the supervised learning back-propagation algorithm in conjunction with optimization technique such as stochastic gradient descent [29, 30]. Regularization techniques, such as Dropout, were introduced to reduce over-fitting and improve generalization [31]. Dropout regularization technique was proposed to address the problem of over-fitting by preventing neurons co-adaption. Recent advances in GPU computing capabilities has allowed complex deep learning architecture to be trained in reasonable amount of time [32].

Lusci et al. [33] were among first to investigate the application of deep neural architectures for modeling molecular properties. The authors utilized a recursive deep neural network approach to model molecular properties and applied the proposed approach to develop predictive model for aqueous solubility. Xu et al. [34] applied deep learning to model drug-induced liver injury. The authors developed a predictive model based on 475 drugs and applied on external dataset of 198 drugs achieving AUC of 0.955 and exceeding the performance of previously reported drug-induced liver injury models. Aliper et al. [35] applied deep learning for predicting pharmacological properties of drugs and for drug repurposing utilizing transcriptomic data from the LINCS Project. The authors demonstrated that DNNs trained on transcriptional response data sets could be utilized to classify drugs indications based on their transcriptional profiles. More recently, attempts to utilize convolutional neural networks (CNN) to model chemical data were also reported. Duvenaud et al., reported a CNN approach that operates directly on molecular graphs, where molecular structures are represented as graphs of arbitrary size and shape [36]. CNN were reported to be able to learn neural graph fingerprints and even outperform standard circular fingerprints on several tested datasets.

Recent study by Ma et al. [19] investigated and compared the performance of deep neural networks to random forest for QSARs applications. The authors employed arbitrary selected DNNs configurations as opposed to an exhaustive search of possible hyper-parameters combinations, and compared the performance of DNNs against Random Forest for regression using 15 Merk’s *in*-*house* datasets including on-target and absorption, distribution, metabolism, and excretion (ADME) endpoints relevant to pharmaceutical research. The authors reported that over 50 DNN configurations were explored and the performance of DNN was compared to RF. DNN was reported to outperform RF overall by achieving on average 0.051 better *R* ^2^ across the 15 activity classes employed.

While several studies utilizing deep learning techniques have been recently reported in the literature, the use of customized *in*-*house* implementations, often based on Theano framework, and models often remain non accessible to readers, hence hindering wider adoption of deep learning techniques [37]. Instead, in this work we opted for the open-source Caffe deep learning framework as the main framework for training and testing deep neural networks configurations. Caffe provides an easy usable platform for defining DNN configurations and executing experiments without extensive customizable hard-coding required as it provides an expressive and highly modular architecture [38]. The code used to carry out this study, which was built upon Caffe and termed “*ChemoCaffe*”, is also made publicly available in the hope to make deep learning more accessible to practitioners interested applying DNNs to chemo-biological problems. *ChemoCaffe* allows large number of arbitrary selected DNN configurations to be tested in an automated fashion until optimal set of hyper-parameter values is identified.

Moreover, there is lack of comprehensive comparison of deep learning methods to popular methods of commonly employed algorithms such as random forest, support vector machine, k-nearest neighbor and Naïve Bayes for modeling structure–activity relationships (SARs). In this study we address this gap in the literature by conducting and reporting a comprehensive comparison utilizing seven diverse bioactivity classes extracted from ChEMBL public repository. Furthermore, it is commonly assumed that there is a limited amount of noise present in bioactivity datasets. There is no empirical evaluation of deep learning methods when dealing with noisy data, a common situation prevalent in Cheminformatics applications, especially when dealing with high-throughput screening readings. Hence, we explored the robustness of machine learning algorithms included in this study by introducing different levels of artificial noise to the datasets and measuring the performance of each method (Table 1).

**Table 1:** Bioactivity datasets assembled from ChEMBL repository and utilized in this study

Activity class	CHEMBL target id	Number of active inhibitors	Number of decoys
Carbonic anhydrase II, Class: enzyme, lyase	CHEMBL205	1631	16,310
Cyclin-dependent kinase 2, Class: protein kinase	CHEMBL301	705	7050
HERG, Class: Voltage-gated ion channel	CHEMBL240	700	7000
Dopamine D4 receptor, Class: membrane receptor, GPCR	CHEMBL219	506	5060
Coagulation factor X, Class: enzyme, serine protease	CHEMBL244	1144	11,440
Cannabinoid CB1 receptor, Class: membrane receptor, GPCR	CHEMBL218	1911	19,013
Cytochrome P450 19A1, Class: enzyme, cytochrome P450	CHEMBL1978	621	6210

The ratio of decoys/active per activity class was set to 10:1

## Results and discussion

The experimental process followed consisted of two main parts. First, arbitrary but reasonable selected hyper-parameter configurations were explored with the aim to investigate how they affect the performance of feed-forward deep neural networks. In the second part of the study, comparison of performance of deep neural nets to shallow methods Bernoulli Naïve Bayes, k-nearest neighbor, random forest and support vector machine was investigated.

### Optimizing deep neural networks hyper-parameters

Here the study was focused on the hyper-parameters: (a) activation functions, by comparing the performance of rectified linear unit (ReLU), Sigmoid (Sigm) and Tanh functions, (b) learning rate, (c) number of neurons per layer, (d) number of hidden layers and (e) dropout regularization. All tested network configurations were trained for 300 epochs with fixed learning rates, as shown in Table 2, with no early stops applied.

**Table 2:** Hyper-parameters values explored for Bernoulli Naïve Bayes, k-nearest neighbor, random forest, support vector machines and deep neural networks

Hyper-parameters	Values explored	Parameter
Bernoulli Naïve Bayes		
Alpha	1, 0.5, 0.1	Laplace/Lidstone smoothing parameter
Fit_prior	True, false	Class prior probabilities. In case of false, a uniform prior was used
k-Nearest neighbor		
Nn	1, 3, 5, 7, 9, 11	Number of nearest neighbors
Random forest		
Ntrees	10, 50, 100, 300, 700, 1000	Number of trees
Criterion	Gini, entropy	Functions used to measure the quality of each split
Max_features	Sqrt(n_features), log2(n_features)	Number of features considered for each split
Support vector machines		
Kernel	rbf	Radial basis function
C	10^3^, 10^2^, 10, 1	Cost
γ	10^−5^, 10^−4^, 10^−3^, 10^−2^, 10^−1^	Gamma
Kernel	Linear	Linear kernel
C	10^3^, 10^2^, 10, 1, 10^−1^, 10^−2^, 10^−3^, 10^−4^	Cost
Deep neural networks		
η	1, 10^−1^, 10^−2^, 10^−3^, 10^−4^	Learning rate for the stochastic gradient descent (“SGD”)
Momentum (μ)	0.9	Weight of the previous update
Weight decay	0.0005	
Epochs	300	Number of training epochs
Batch size	256	mini-batch training size
Hidden layers	1, 2, 3, 4	Number of hidden layers
Number neurons	5, 10, 50, 100, 200, 500, 700, 1000, 1500, 2000, 2500, 3000, 3500	Number of neurons per hidden layer
Activation function	ReLU, Sigmoid, Tanh	Neuron activation functions
Regularization	No, Dropout	Regularization techniques
Dropout	(0%, 20%, 50%) input layer, 50% hidden layers	% of neurons “dropped” using the Drop-out technique
Weight and bias initiation	Gaussian {SD: 0.01}	Function used to initiate weights and biases.
Loss function	SoftmaxWithLoss	Function used to minimize loss
Output function	Softmax	Function used to calculate probability for predictions
Number of classes	2	Binary classification

#### Comparing activation functions

**Fig. 2:**
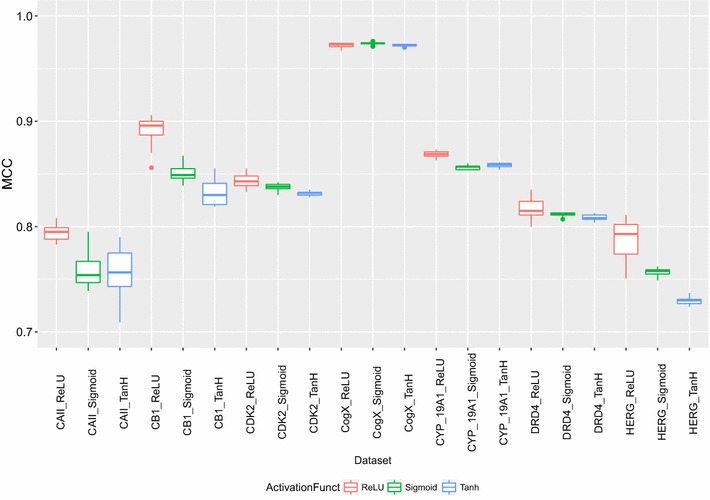
Comparison of activation functions rectified linear units (ReLU), Tanh and Sigmoid (Sigm) on the performance of DNNs. DNNs with a single hidden layer and variable number of neurons were trained and tested using the activation functions ReLU (*red*), Sigm (*green*) and Tanh (*blue*) over fivefold cross validation. The performance was measured using MCC as evaluation metric

Single hidden layered neural networks with varying number of neurons {5, 10, 50, 100, 200, 500, 700, 1000, 1500, 2000, 2500, 3000, 3500} were tested to compare the performance between the three activation functions ReLU, Sigm and Tanh, the rest hyper-parameters were kept fixed. The performance was measured using MCC over fivefolds cross validation. The results obtained per activity class and per activation function are presented in Fig. 2 with ReLU(red), Sigmoid (green) and Tanh (blue), obtained with learning rate of *η* = 0.1. Neural Networks combined with the ReLU activation function perform better, when compared to Sigm or Tanh, by achieving higher MCC. Statistical analysis using Wilcoxon paired rank test are shown in Table 3, with confidence interval 99%. The results demonstrate that DNN combined with the rectified linear units (ReLU) activation function performed better than the Sigm or Tanh functions. Neurons with rectified linear units (ReLU) activation function has been reported to be (a) easier to optimize, (b) converge faster, (c) generalize better and (d) are faster to compute [39].

**Table 3:** Pairwise comparison of performance between deep neural networks with rectified linear units (ReLU) against Sigmoid (Sigm) and Tanh activation functions based on the Wilcoxon paired signed rank test, with confidence intervals 99%

Activation functions	Mean of MCC diff.	SD of MCC diff.	*p* value
ReLU—Sigm	0.018	0.022	3.922e−11
ReLU—Tanh	0.029	0.033	3.417e−14

DNN combined with ReLU function were found to statistically outperform Sigm and Tanh functions

#### Number of hidden layers, neurons per layer and regularization

Feed-forward neural networks are considered “deep” when the number of hidden layers is equal to or more than two, where each layer represents a higher level of abstraction. Here the analysis was focused on the hyper-parameters; (a) number of hidden layers, (b) learning rate, (c) number of neurons per hidden layer and (d) “dropout” regularization, while retaining the rest hyper-parameters fixed. Experimental process followed was similar as before using internal fivefold cross validation.

**Fig. 3:**
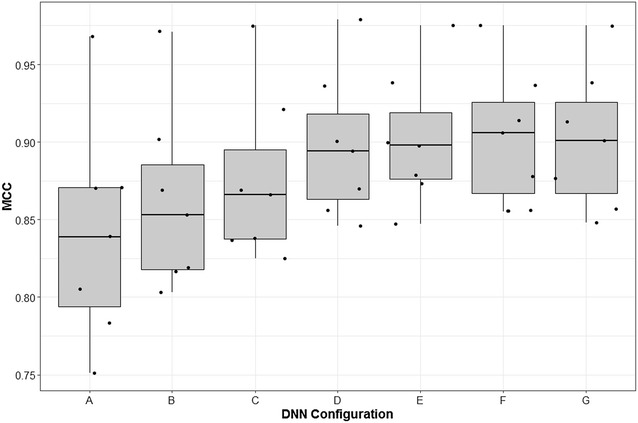
Effect of the hyper-parameters (i) number of hidden layers, (ii) number of neurons and (iii) dropout regularization on the performance of DNNs measured by MCC as evaluation metric. DNN configuration *A* shows results obtained by DNN with a single hidden layer and 10 neurons, ReLU activation function and no regularization averaged over the seven activity datasets, *B* a two hidden layered DNN with 500 neurons in each layer, ReLU activation function and no regularization, *C* two hidden layers with 3000 neurons per hidden layer and dropout regularization (0% for the input and 50% for hidden layers), *D* two hidden layers with 3000 neurons per hidden layer and dropout regularization (20% for the input and 50% for hidden layers), *E* two hidden layers with 3000 neurons per hidden layer and dropout regularization (50% for both the input and hidden layers), *F* three hidden layers with 3000 neurons per layer and dropout regularization (50% for both the input and hidden layers) and *G* four hidden layers with 3500 neurons per layer and dropout regularization (50% for the input and hidden layers)

Results obtained by seven deep neural nets configurations over the seven bioactivity classes are shown in Fig. 3, here the effect of hyper-parameters (a) number of hidden layers, (b) number of neurons and (c) dropout regularization on the performance of DNN measured by MCC as evaluation metric are visualized averaged over the seven activity classes, while the rest parameters were kept fixed. From these results it can be observed that increase of the number of hidden layers and neurons, combined with application of regularization, has a substantial effect on the performance of DNN. When comparing the improvement in performance observed between single hidden layered Neural Networks, configuration A, and deeper network configurations tested (B–G) with multiple hidden layers, thousands of neurons and regularization the performance was improved by an average of 0.062 MCC units. Furthermore, application of dropout regularization on the performance, configurations C–G, was also found to affect the performance, when comparing to non-regularized networks. As an example, the performance was improved by an average of 0.039 MCC units when dropout of 50% was applied to two hidden layered DNN. This highlights the importance of regularization on the performance of DNN.

### Comparison of performance of deep neural nets to shallow methods and robustness to noise

In the second part of the study comparison of performance of deep neural nets with the shallow counterparts Bernoulli Naïve Bayes (NB), k-nearest neighbor (kNN), random forest (RF) and support vector machines (SVM) was performed. Here the NB classifier was used as a baseline method. Each dataset was split to 60% for hyper-parameters tuning using fivefold cross validation and the rest 40% was used as validation set, with the experiments repeated three times for each dataset. Performance achieved by each algorithm on validation sets is presented in Table 4. Wilcoxon paired signed-rank statistical test was applied, with confidence intervals 99%, to compare the performance achieved by DNN against each of the rest methods, results are shown in Table 5.

**Table 4:** Performance achieved by DNN, NB, kNN, RF and SVM measured using MCC as evaluation metric

Dataset	Algorithm	MCC 1st	MCC 2nd	MCC 3rd	Mean MCC	Std MCC
DRD4	DNN	*0.900*	*0.895*	*0.867*	*0.887*	0.018
	SVM_rbf	0.889	0.876	0.865	0.876	0.012
	SVM_linear	0.828	0.816	0.840	0.828	0.012
	RF	0.867	0.854	0.862	0.861	0.007
	kNN	0.762	0.778	0.763	0.767	0.009
	NB	0.742	0.761	0.750	0.751	0.009
HERG	DNN	*0.858*	*0.913*	*0.880*	*0.884*	0.028
	SVM_rbf	0.845	0.891	0.872	0.869	0.023
	SVM_linear	0.773	0.782	0.780	0.778	0.005
	RF	0.838	0.857	0.848	0.847	0.010
	kNN	0.818	0.813	0.825	0.819	0.006
	NB	0.612	0.620	0.602	0.611	0.009
CDK2	DNN	0.919	*0.932*	*0.927*	*0.926*	0.007
	SVM_rbf	*0.920*	0.913	0.922	0.919	0.005
	SVM_linear	0.863	0.889	0.864	0.872	0.015
	RF	0.895	0.912	0.902	0.903	0.008
	kNN	0.895	0.904	0.910	0.903	0.007
	NB	0.769	0.773	0.780	0.774	0.006
CogX	DNN	*0.982*	*0.981*	*0.987*	*0.983*	0.003
	SVM_rbf	0.978	0.979	0.980	0.979	0.001
	SVM_linear	0.971	0.970	0.977	0.973	0.004
	RF	0.973	0.979	0.982	0.978	0.004
	kNN	0.968	0.971	0.971	0.970	0.002
	NB	0.889	0.897	0.882	0.889	0.008
CYP_19A1	DNN	*0.893*	*0.920*	*0.899*	*0.904*	0.014
	SVM_rbf	0.886	0.896	0.889	0.890	0.005
	SVM_linear	0.849	0.866	0.862	0.859	0.009
	RF	0.873	0.910	0.879	0.887	0.020
	kNN	0.805	0.811	0.821	0.812	0.008
	NB	0.755	0.821	0.775	0.784	0.034
CB1	DNN	*0.943*	*0.941*	*0.940*	*0.941*	0.002
	SVM_rbf	0.941	0.937	0.931	0.936	0.005
	SVM_linear	0.885	0.893	0.881	0.886	0.007
	RF	0.908	0.923	0.914	0.915	0.008
	kNN	0.906	0.921	0.901	0.909	0.011
	NB	0.758	0.781	0.765	0.768	0.012
CAII	DNN	*0.858*	*0.885*	0.843	*0.862*	0.021
	SVM_rbf	0.857	0.851	*0.866*	0.858	0.007
	SVM_linear	0.828	0.826	0.830	0.828	0.002
	RF	0.836	0.857	0.861	0.851	0.013
	kNN	0.558	0.557	0.577	0.564	0.011
	NB	0.754	0.769	0.783	0.769	0.015

Results for each activity class and validation set from three experiments as shown. Best recorded results for each activity class are highlighted in italic

**Table 5:** Wilcoxon paired signed-rank test was employed to compare the performance of DNN against the rest algorithms NB, kNN, RF and SVM across the datasets, with confidence intervals 99%

Algorithms	Mean MCC diff.	SD of MCC diff.	*p* value
DNN-NB	0.149	0.061	4.768E−07
DNN-kNN	0.092	0.095	4.768E−07
DNN-SVM (linear)	0.052	0.031	3.2E−5
DNN-RF	0.021	0.016	8.6E−5
DNN-SVM (rbf)	0.009	0.012	5.075E−4

Below are reported the means and standard deviations of the observed MCC for each algorithm and the *p* value

**Fig. 4:**
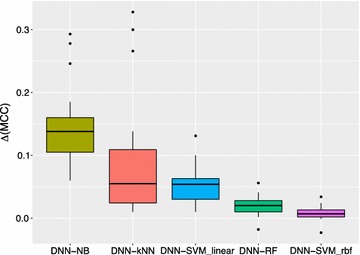
Boxplot of differences between performances achieved by tuned DNN and the rest algorithms measured using MCC as evaluation metric on the validation sets over the seven activity classes. Results are ranked by decreased mean differences. The differences ranged on average from 0.149 MCC units between DNN and NB, 0.092 DNN and kNN, 0.052 DNN and SVM with linear kernel, 0.021 DNN and RF and 0.009 DNN and SVM with “rbf” kernel

The differences measured in MCC units between DNN and the rest algorithms are visualized in boxplot Fig. 4. Here DNN achieved on average MCC units of 0.149 higher than NB, 0.092 than kNN, 0.052 than SVM with linear kernel, 0.021 than RF and finally 0.009 higher than SVM with radial basis function kernel. These results demonstrate that deep neural nets, when tuned accordingly, are capable of achieving strong performance for modeling bioactivity data for small molecules and outperform popular machine learning methods when combined with simple circular fingerprints as molecular descriptors.

**Fig. 5:**
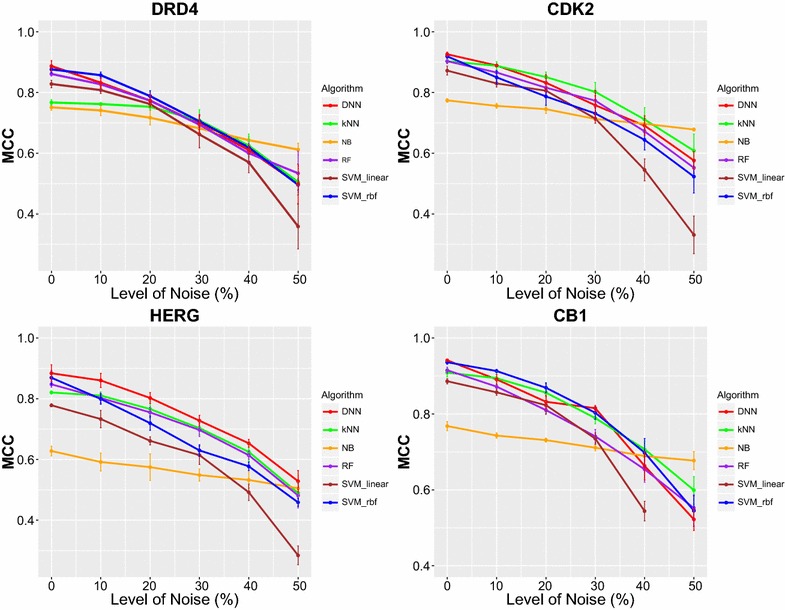
Robustness of machine learning methods to different levels of noise for 4 out of 7 activity classes. At low levels of noise, lower that 20%, non-linear methods performed well achieving performance higher than 0.7 MCC units for most of the tested datasets. Instead, at higher level of noise, equal to or higher than 30%, performance for most algorithms dropped below 0.7 MCC and in several occasions even lower than 0.6 at 50% of noise. Naïve Bayes method was found to be the least affected method achieving in several tested datasets performance higher than 0.6 MCC even at the highest level of noise tested 50% and outperforming more complex methods

Structure–activity relationships collected from medicinal literature and stored in bioactivity databases may contain noise or erroneous reported activity measurements, due to human error or experimental uncertainty, that could potentially affect the performance of data-mining techniques [40–43]. Furthermore, results from high-throughput screenings (HTS) are known to suffer from false positive hits. Hence, we explored robustness of machine learning algorithms to noise by introducing artificial noise to the datasets and measuring the effect on the performance of the data mining techniques. Noise was introduced by randomly sampling and flipping the labels of a percentage of active instances onto inactive and vice versa equal number of randomly sampled inactive instances to active, in total five levels of noise were introduced in each activity dataset, ranging from 10 and up to 50%. This process was applied on the training sets, while the validation sets were not altered to serve as ground truth when measuring performance. The results from the process for 4 of the dataset are presented in Fig. 5. When examining robustness of machine learning algorithms to different levels of noise it was observed that non-linear methods overall outperformed Naïve Bayes when dealing with low levels of noise, lower or equal to 20%, where performance was retained equal to or higher than MCC of 0.7 on average. Instead, at higher level of noise equal or higher than 30% Naïve Bayes reached or even outperformed the rest methods, e.g. in cases of datasets CDK2 and CB1, demonstrating higher tolerance to noise. Naïve Bayes has been previously reported to perform well when dealing with noisy high-throughput screening data and achieve good enrichments among top retrieved compounds [44]. DNN overall outperformed the rest methods across most tested datasets, when dealt with noisy data their performance in several datasets dropped below that of other methods included in the study indicating that these methods might be more sensitive to noise that the rest methods. Hence when dealing with experimental datasets where low level of noise is expected non-linear methods are likely be a better option, e.g. measurements generated from confirmatory screening campaigns, instead when dealing with data where high level of noise is expected, e.g. high-throughput screening campaigns, methods such as Naïve Bayes might be of value.

## Conclusions

In this study feed-forward deep neural networks were investigated for modeling bioactivity data. The aim of the study was dual, first DNN hyper-parameters values were explored to investigate how they affect the performance of feed-forward DNN. Below we summarize and compare our findings with those reported by Ma et al. [19], where strong agreement were observed:

- Rectified linear units (ReLU) activation function performed overall better than Sigmoid or Tanh. Hence, it is highly recommended to use ReLU activation function when training DNN.
- The number of hidden layers should be at least 2 or 3 in order for DNN to achieve strong performance. Further increase of number of hidden layers had diminishing returns as best results were obtained when using 2 or 3 hidden layers.
- The number of neurons per hidden layer was found to depend on the dataset and should be optimized on a case-by-case basis. While it’s recommended to use at least 100 neurons in each hidden layer, best results were obtained when using larger number of neurons, e.g. 700, 1000, 2000 or higher.
- Dropout regularization technique was found to have great impact on the performance of DNN by preventing co-adaption of neurons. Here it was found that dropout should be applied to both input and hidden layers. Good performance was achieved when dropout of 50% was applied to both input and hidden layers.
- No pre-training of DNN was performed and both the weights and biases were randomly initialized from Gaussian distribution. Similarly, Ma et al. reported that no unsupervised pre-training is needed and network parameters should be initialized as random values.
- The number of epochs should be as many as possible, at least 300, given the local computing capability. Modern GPU units provide adequate computational power to optimizing DNN configurations in reasonable time. Combined with Dropout technique over-fitting shouldn’t be a major concern.

In the second part of the study, the performance of DNNs was compared to widely popular shallow methods Bernoulli Naïve Bayes, k-nearest neighbor, random forest and support vector machines. Here it was found that optimized deep neural nets achieved statistically better performance, measured by the Wilcoxon test on the tested datasets. These results demonstrate the deep-learning techniques can serve as powerful modeling techniques for modeling complex biological data. While deep learning techniques are highly unlikely to replace existing algorithms any time soon, their popularity in the field of Cheminformatics is expected to grow as the availability of GPU hardware and of stable and well-documented software packages become more accessible to Cheminformatic practitioners.

Interpretation of local and global SAR captured by complex QSAR models remains a challenging task, in particular when nonlinear algorithms are employed [45]. Several approaches in the literature have been proposed for interpreting local and global QSAR models based on support vector machine and random forest techniques [46, 47]. DNNs are generally considered as “black boxes” and are difficult to be interpreted [15]. Hence, future studies should aim to investigate how models learned by DNN could be interpreted, as this could help rationalizing structure–activity relationships encoded by models. This knowledge could drive the lead optimization process by suggesting structural modifications that might be beneficial for the primary activity, while decreasing any undesired off-target interactions.

## Methods and Materials

### Bioactivity datasets

As source of structure–activity relationships (SARs) the ChEMBL database was employed [48, 49]. ChEMBL, version 20, was used in this study. In total seven diverse bioactivity classes were selected and used in the study: (a) Carbonic Anhydrase II (ChEMBL205), a protein lyase, (b) Cyclin-dependent kinase 2 (CHEMBL301), a protein kinase, (c) ether-a-go-go-related gene potassium channel 1 (HERG) (CHEMBL240), a voltage-gated ion channel, (d) Dopamine D4 receptor (CHEMBL219), a monoamine GPCR, (e) Coagulation factor X (CHEMBL244), a serine protease, (f) Cannabinoid CB1 receptor (CHEMBL218), a lipid-like GPCR and (g) Cytochrome P450 19A1 (CHEMBL1978), a cytochrome P450. The activity classes were selected based on data availability and as representatives of therapeutically important target classes or as anti-targets.

SARs data were extracted following the criteria: (a) Only human direct protein targets were considered, (b) confidence score equal to 9, (c) MW up to 900, (d) activity was defined based on pKi or pIC50, depending which type of measurement was the majority of available per activity class, (e) active compounds were defined those with potency, pKi or pIC50, better than or equal to 5, which equals to or better than 10 μM. Decoys were randomly sampled from a large pool of drug-like compounds extracted from ChEMBL, covering small bioactive molecules reported against protein targets with potencies (Ki/IC50/Kd) up to 10 μM and MW up to 900. As an additional step chemical structures with Tanimoto similarity coefficient larger than 0.9 were removed. For each active structure, 10 decoys were randomly sampled. The main motivation here was to assemble reasonably sized datasets for each activity class, suitable for comparing the performance of the machine learning methods included in the study while avoiding creating highly unbalanced datasets. Number of active and decoys assembled per activity class is shown in Table 1.

Chemical structures were standardized using the MOE software package with the options on: (a) disconnect group/metals in simple salts, (b) keep only largest molecular fragments, (c) deprotonate strong acids, (d) protonate strong bases and (e) replace coordinates with a generated 2D depiction [50]. As molecular descriptors the Morgan fingerprints as implemented in the Rdkit Cheminformatic toolkit were utilized with radius 2, which are equal to extended connectivity fingerprints (ECFP_4), and stored in hashed 1024 bit length binary vectors [51, 52]. ECFP fingerprints were selected for this study as they have been previously shown to perform well and are commonly employed in virtual screening applications [53]. The datasets utilized in this study are provided in Additional file 1.

### Machine learning methods

#### Bernoulli Naïve Bayes

The first of the shallow algorithms employed in this study was the Bernoulli Naïve Bayes classifier (NB) [54]. Naïve Bayes classifier is a probabilistic supervised machine-learning algorithm based on the Bayes’ theorem with the strong “naive” assumption of feature independence [54–56]. NB was included as a baseline method in this study as implemented in the Scikit-learn library utilizing the “BernoulliNB” function [57]. The explored hyper-parameters values are presented in Table 2.

#### k-Nearest neighbor

The second algorithm employed was the k-nearest neighbor (kNN) [58]. K-Nearest Neighbor was utilized as implemented in the Scikit-learn library. Here the “KNeighborsClassifier” function was employed. Only the hyper-parameter number of nearest neighbors was explored while the rest were retained default. The explored hyper-parameters values are presented in Table 2.

#### Random forest

The third algorithm employed was the random forest (RF). RF is an ensemble of multiple weak un-pruned classification or regression trees created by using bootstrap samples of the training data and random feature selection in tree induction [59]. RF was developed by Breiman and Cutler [60] and presents a number of advantages, which makes it attractive and well suited for cheminformatic applications; (a) it is robust when dealing with large number of features, (b) achieves generally good performance, (c) resilient to over-fitting and (d) can be parallelized across multiple CPU cores. Furthermore, it can be employed to assess the relevance of features, effectively acting as a feature selection algorithm [61, 62]. RF as implemented in the scikit-learn library was employed, “*RandomForestClassifier*” function from the ensemble family [57]. The explored hyper-parameters values are presented in Table 2.

#### Support vector machines

The fourth algorithm employed was the support vector machines (SVMs). SVMs along with RF is a widely applied machine-learning algorithm in the field of Cheminformatics [63, 64]. SVMs was proposed by Cortes and Vapnik and can be applied for classification, regression and outliers detection tasks [65]. In this study, two kernels were considered the non-linear radial-basis function and the linear kernel. SVM as implemented in the scikit-learn library was employed. Scikit-learn implementation of SVM is based on the Libsvm and the Liblinear libraries [66, 67]. When optimizing the SVM using the non-linear RBF kernel the values for hyper-parameters gamma (*γ*) and Cost (*C*) where selected with similar range to those reported by Alvarsson et al. [68]. Here values for gamma (*γ*) tested were 10e−1, 10e−2, 10e−3, 10e−4, 10e−5 and for cost (*C*) 1, 10, 100, 1000. The explored hyper-parameters values are presented in Table 2. Results obtained when optimizing the SVM algorithm using the RBF kernel were in good agreement with those reported by Alvarsson et al., where for gamma (*γ*) equal to 0.01 and Cost (*C*) values of 10, 100 and 1000 the highest values in performance were obtained across all datasets.

#### Deep neural networks

As mentioned earlier the Caffe deep learning framework was adopted in this study. In order to automate the process and investigate multiple hyper-parameters configurations the Python wrapper “ChemoCaffe_tune.py” and “ChemoCaffe_predict.py” were developed, which are built upon the pyCaffe module. Caffe requires the input data to be either in *LMDB* [69] or *HDF5* [70] format; we opted for the *HDF5* format mainly due to convenience. No feature transformation or pre-training of DNNs was employed in this study. DNNs were optimized on a dedicated server with NVIDIA Tesla K40 GPU units.

The hyper-parameters selected and explored for DNNs were: (A) Default parameters, those that were kept fixed across all tested configurations: (a) As optimization method the *Stochastic Gradient Descent* (“*SGD*”) with momentum *μ* = 0.9 was used, (b) number of epochs was set to 300, (c) mini-batch size of 256, (d) Weights and biases were initialized from Gaussian distribution with Standard deviation of 0.01 and (e) As loss function the logistic softmax was used, “*SoftmaxWithLoss*” and (d) softmax as the output function for calculating class probabilities and (g) the weight_decay was set to 0.0005. (B) Variable hyper-parameters: (a) Activation functions compared the rectified linear units (ReLU), Sigmoid (“sigm”) and Tanh (“tanh”), Fig. 1c illustrates the shapes of the functions. (b) Number of neurons in each hidden layer (5, 10, 50, 100, 200, 500, 700, 1000, 1500, 2000, 2500, 3000, 3500), (c) learning rate “*η*” of (1, 10^−1^, 10^−2^, 10^−3^, 10^−4^), (d) number of hidden layers up to 4, (e) regularization technique applied; (1) no regularization and (2) dropout. In case of dropout regularization: for input layer dropout of 0, 20 and 50% were applied and for the hidden layer of 50%. The explored hyper-parameters values are presented in Table 2. The python wrappers for tuning hyper-parameters “*ChemoCaffe_tune.py*” and generating predictions “*ChemoCaffe_predict.py*” used to carry out this study as also the configuration files containing the tested configurations are provided in Additional file 2.

### Performance metric

#### Matthews correlation coefficient (MCC)

As performance evaluation metric the Matthews correlation coefficient (MCC) was utilized to measure and compare the performance of machine learning algorithms employed in the study [71, 72]. MCC is a commonly applied performance evaluation metric to measure the quality of binary classification and can be calculated according to the Eq. (1). It measures the correlation between predicted and the actual class labels. MCC is generally considered to be a balanced evaluation metric and takes values between −1 and +1, where −1 indicates perfect anti-correlation between predicted and real observations, +1 represents perfect prediction and 0 that equals to no better than random,

1 $$MCC = \frac{TP \times TN - FP \times FN}{{\sqrt {\left( {TP + FP} \right)\left( {TP + FN} \right)\left( {TN + FP} \right)\left( {TN + FN} \right)} }}$$

where TP are true positives, TN true negatives, FP false positives and FN false negatives (Additional file 3).

#### Wilcoxon signed-rank test

Wilcoxon paired singed-rank test a non-parametric statistical hypothesis test was used to compare matched samples to assess whether their population mean ranks differ [73]. Wilcoxon test as implemented in the R programming language was utilized, part of the “*stats*” package, with confidence interval 99% and alternative hypothesis being “*greater*” [74].

## Additional files

**Additional file 1.** Raw data used in this project.

**Additional file 2.** Code developed in this project.

**Additional file 3.** Results of robustness testing.

**Additional file 4.** Results of parameter selection.
